# Digital divide and the health of internal elderly migrants in China: A cross-sectional study

**DOI:** 10.1371/journal.pone.0305655

**Published:** 2024-07-08

**Authors:** Yuping Liu, Ruixi Wang, Junjun Guo

**Affiliations:** 1 Institute of Public Policy, China West Normal University, Nanchong, Sichuan, China; 2 School of Politics and Administration, China West Normal University, Nanchong, Sichuan, China; Flinders University, AUSTRALIA

## Abstract

**Introduction:**

Population aging and internal migration have become the "norm" in China’s population development. Influenced by both "mobility" and "old age," internal elderly migrants (IEMs) face the second-level digital divide problems primarily characterized by digital technology usage gap, which can lead to adverse health outcomes. Understanding the impact of the digital divide on the health of IEMs can provide effective solutions to meet the health needs of this particular group and facilitate their better integration into a digital society. Therefore, this study aims at exploring the impact of the digital divide on the health of IEMs, and identifying priorities and recommendations for improving IEMs’ health by mitigating the adverse effects of the digital divide.

**Methods:**

In the 2017 China Migrant Dynamic Survey (CMDS), a cross‑sectional sample of 169,989 internal migrants in 32 provincial units across China was recruited by stratified probability proportionate to size sampling (PPS). We focus on IEMs and require interviewees to be 60 years and older. Therefore, we excluded samples younger than 60 years of age and retained only 6,478 valid samples. Subsequently, STATA 17.0 software was applied to analyze the data. Based on the research objective and Grossman’s model of health demand, we empirically tested using ordered logit regression.

**Results:**

The digital divide does affect the health of IEMs in general and its negative effects tend to decrease significantly with age. In terms of groups, its impact showed noticeable group differences in residence arrangement, public health services and medical insurance coverage. Compared with IEMs who live alone or only live with their spouse, have not received public health service, and have not been covered by any medical insurance, the digital divide imposes a smaller adverse impact on the health of IEMs who live with at least one offspring, have received public health service, and have covered in at least one medical insurance. In terms of potential mechanisms, among the effects of digital divide on the health of IEMs, the mediating effect of urban integration is not significant, the social interaction has only a partial mediating effect, and the medical convenience has a significant mediating effect.

**Conclusion:**

Our findings confirm the existence of the third-level digital divide among IEMs concerning health, that is, the digital divide has adverse health outcomes for this group, and underscore the important implications of reducing the negative impact of the digital divide in improving the health status of IEMs.

## Introduction

Since the 2016 Outline of the Healthy China 2030 Plan called for improving people’s health, the 19th Communist Party of China National Congress further made important decisions to advance the Healthy China Initiative, which elevated safeguarding people’s health as a national strategy. In the critical period of building a healthy China, the demographic situation is also undergoing complex and profound changes, with aging and internal migration of population gradually becoming a norm for China’s population development in the new era. In this context, internal elderly migrants (IEMs) have captured considerable attention from all walks of life due to their dual characteristics of mobility and old age [1]. China’s IEMs reached 1.78 million in 2020, accounting for 7.2 percent of the total internal migrants and are expected to continue growing in the future, according to the National Health Commission’s Report on China’s Migrant Population Development in 2021.

With the development of the Internet and information technology, the digital economy has played an increasingly important role in the Chinese economy and society in recent years. Digital opportunities and dividends related to digital economy are transforming people’s production and lifestyle, as well as traditional social structures and forms with unprecedented momentum [2]. However, some groups may be actively or passively excluded from the digital society due to factors such as technology, systems, and culture in the rapid digitalization process [3], leading to the issue of the digital divide [4]. The digital divide was initially defined as the binary disparity between individuals who have access to Information and Communications Technology (ICT) and those who do not, representing a divide in digital access (the first-level digital divide) [5]. As research progressed, some scholars found that people with Internet access have great differences in the way the digital technology usage, leading to the emergence of a second-level digital divide [6]. The digital divide is evident not only in the dimensions of ICT, but also in the diverse outcomes that arise from the use of digital resources by different groups in social life [7], giving rise to the concept of the third-level digital divide [8]. The digital divide not only prevents people from equally enjoying the development achievements of information transmission, social communication, and medical services, but also adversely affects the digitally disadvantaged people in multi-dimension, even turning them into digital refugees in the new era [9].

Influenced by the dual impact of mobility and old age, IEMs not only have unique health demands, but are also more likely to be eroded by the digital divide, making them especially prone to becoming a national health shortcoming in China. Currently, few researchers are discussing the impact of digital divide on IEMs’ health. However, several scholars have paid attention to the impact factors on the health of IEMs [1,10,11]or tested the health effect of digital divide [12,13]. In the digital era, however, it is essential to clarify the relationship between the digital divide and the health of IEMs for whether and how to improve their overall health by bridging the digital divide.

This article examines the impact of the digital divide on IEMs in China from a health perspective, analyzing the heterogeneity of the effect, and further exploring the its mechanisms. Compared with existing studies, this paper has two innovative aspects: first, it broadens research on the third-level digital divide in terms of both the research object (taking IEMs with the dual characteristics of mobility and old age as the research object) and research perspective (from a health perspective) to discusses the consequence of digital technology usage gap; Second, from the standpoint of reducing the adverse effects of digital divide, this paper proposes policy proposals to improve the overall health level of IEMs in China and then put their health security into the promotion of digital economic development, which is in line with the current policy background of the Healthy China Initiative and the realistic environment of digital economic development. We expect that this paper will provide decision-making references and scientific advice for formulating and complementing health security policies for the elderly from the perspective of mitigating the adverse effects of the digital divide.

## Methods

### Data source

The article employs data from the 2017 China Migrant Dynamic Survey (CMDS) to ensure that the data are scientific and representative. CMDS is an annual large-scale nationwide dynamic survey of the migrant population conducted by the National Health Commission of China since 2009, adopting a stratified probability proportionate to size sampling (PPS) method as the sampling technique. The survey samples include 31 provinces and Xinjiang Production and Construction Corps in mainland China, focusing on areas with a high concentration of internal migrants. Its annual sample size of nearly 200,000 households covers basic demographics information of internal migrants and their household members, migration rang and destinations, employment and social insurance, income, expenditure and residence, essential public health services, marriage and family planning services, children’s migration and education, psychological culture, etc.

The object of this study is IEMs in China, a group that possesses both mobility and old age attributes. In CMDS, "mobility" is defined as individuals who separate from the district (county/city) of their household residence for more than one month. "old age" requires that the interviewee be at least 60 years old at the time of survey. In 2017 CMDS, a total sample size of 169,989 migrants was investigated. After excluding samples that were under 60 years old, a total of 6,478 valid samples were retained for this study.

### Measurements

#### Dependent variable

Self-rated health status is a subjective evaluation made by individuals based on their physical health status, which can comprehensively reflect the overall physical and mental health status of people to a certain extent, and it is widely used in various studies on health assessment and influencing factors [14]. Therefore, the self-rated health status of IEMs is used as the dependent variable to characterize their health status. In 2017 CMDS, the question about the self-rated health status of migrants is "How is your health status" and the answers from options 1 to 4 are"1 = healthy, 2 = basically healthy, 3 = unhealthy (but able to take care of themselves), 4 = disable (unable to take care of themselves)". That is, the higher the number, the worse the health status.

#### Independent variables

To study the health consequences caused by the digital technology usage gap, independent variables should be pointed at the usage gap in digital technology. While the Internet is a wealth of health-related information, and all levels of health insurance institutions conduct health education online, not all IEMs have the same level of ability to access this information. Some are more receptive and skilled in using digital technology, while others are not. Therefore, digital technology usage ability (i.e. skills to access network health education information) is assessed by the question "How did you receive the above health education in your current village/community?" We assign a value of 0 to IEMs who get health education information via Community SMS/Wechat/Website (indicating strong ability), and a value of 1 to those who do not through these channels (indicating weak ability). This allows us to construct a binary discrete variable of digital technology usage ability as the independent variable.

#### Control variables

Referring to the related literature on the factors affecting the health of migrant populations [15], the control variables mainly involve two types, i.e., (1) individual characteristics, including gender, age, ethnicity, education, hukou status, as well as marital status; (2) migration characteristics that include migration range, migration duration, and work in inflow place.

#### Heterogeneity variables and mechanism variables

This article performed both heterogeneity and mechanism analyses to further examine the discrepancy in health outcomes caused by digital technology usage ability in different situations and the mechanisms of impact. The heterogeneity variables include residence arrangement, public health services, and medical insurance coverage. social contact, urban integration, and medical convenience are the main mechanism variables. **Table 1** reports the definitions and descriptive statistics of the main variables.

**Table 1:** Definition and descriptive statistics of the main variables.

Variables	Variable Definition	CMDS code	N	Mean/Proportion	SD
**Dependent Variable**					
Self-rated health status	1 = healthy, 2 = basically healthy, 3 = unhealthy (but able to take care of themselves), 4 = disable (unable to take care of themselves in life)	401	6478	1.772	0.773
**Independent variable**					
Digital technology usage ability(*digital*)	For whether get health information via Community SMS/Wechat/Website. 0 = strong (yes), 1 = weak (no)	405-E	3640	0.733	0.442
**Control variables**					
Gender	0 = male, 1 = female	101-B	6478	0.418	0.493
Age	Age at the time of the survey	101-C	6478	66.046	5.606
Ethnicity	0 = Han, 1 = Minority	101-D	6478	0.088	0.284
Education(*edu*)	1 = none, 2 = primary school, 3 = middle school, 4 = high school, 5 = college and above	101-E	6478	2.648	1.118
Hukou status(*hukou*)	0 = rural, 1 = urban	101-F	6478	0.423	0.494
Marital status(*marriage*)	married (first marriage/remarriage/cohabitation) = 0, unmarried (never married/ divorced/widowed) = 1	101-H	6478	0.160	0.366
Migration range(*range*)	1 = inter-provincial, 2 = inter-municipal within province, 3 = inter-county within city	101-L	6478	1.757	0.772
Migration duration(*duration*)	years of current migration	101-M	6478	8.954	8.141
Work in an inflow place(*work*)	0 = yes, 1 = no	201	6478	0.688	0.463
**Heterogeneity variables**					
Residence arrangement(*reside*)	0 = living alone or with a spouse only, 1 = living with at least one offspring	101_K	6478	0.281	0.450
Public health services(*phservice*)	0 = received, 1 = unreceived	407	2270	0.541	0.498
Medical insurance coverage(*insurance*)	0 = uncovered, 1 = covered	504	6478	0.931	0.253
**Mechanism variables**					
Social contact(*contact*)	For how often contact with others. 0 = seldom, 1 = sometimes/often	309	6478	0.630	0.483
Urban integration(*integration*)	For self-perception that local residents whether willing to identify themselves as local people. 1 to 4 indicates an increasing degree of urban integration in turn	503-D	6478	3.416	0.619
Medical convenience (*trouble*)	For whether do not see a doctor due to inconvenience. 0 = yes, 1 = no	409–6	2510	0.548	0.498

### Model

According to Grossman [16], health is a "durable" capital stock—health capital, which is different from other types of human capital. The demand for health can be explained through supply and demand curves for health capital, where the marginal benefits of health (including monetary values and the direct utility derived from health) equate to the marginal costs (including interest, and depreciation). Numerous scholars have conducted substantial empirical research on the factors influencing residents’ health outcomes, drawing on Grossman’s model of health demand. They have discovered that residents’ health is influenced by multiple factors, including medical insurance, healthcare services, educational attainment, household income, and language proficiency [17,18]. Among these, the ability for digital technology usage, as a significant form of human capital, can, directly and indirectly, influence people’s health levels by various pathways, such as enhancing the ability to access health information, facilitating better communication between doctors and patients, and enabling more effective use of medical services.

Based on the research objective and concerning Grossman’s model [16] of health demand, this paper proposes the following empirical model that examines the factors influencing the health of IEMs.


$$health_{i}=\beta digital_{i}+\sum \varphi_{i}x_{i}+u_{i}$$ (1)

Where *health* is the dependent variable indicates self-rated health status, and *digital* is the independent variable indicates digital technology usage ability and its coefficient is *β* (it is also the value that this paper focuses on). The term *x* is a set of control variables, *φ* is the coefficient of the corresponding control variable and *u* denotes the stochastic disturbance term. Because the dependent variable *health* is an ordered discrete choice variable, we use ordered logit regression to estimate parameters in this equation.

All statistical analyses were performed by STATA version 17.0 (StataCorp LLC. Texas, USA).

## Results

### Digital divide and the distribution of self-rated health

According to the 2017 CMDS, almost all respondents have communication devices to access the Internet or receive WeChat/Community SMS, while it may also be deduced that most villages(communities) have performed health publicity and education over the network from the distribution of online health information recipients. It suggests that, in terms of health information accessibility, the gap in access to digital infrastructure (the first-level digital divide) among internal migrants is less. However, among all the respondents, only 40.18% of internal migrants reported receiving health education through Community SMS/WeChat/Website, which manifests a noticeable gap in the digital technology usage ability of internal migrants, that is, compared with the first-level digital divide, internal migrants face a more significant second-level digital divide. Moreover, the proportion of samples for IEMs receiving health education through Community SMS/WeChat/Website further decreased to 26.68%, confirming that IEMs confront a wider second-level digital divide than younger generations.

From the distribution of self-rated health status (**Table 2**), it is apparent that while most IEMs report "healthy" (42.74%) or "basically healthy"(38.51%) in self-rated health status, the combined proportion of the two, particularly the "healthy" rating, is significantly lower than that of the internal migrants(82.18%). It suggests that IEMs are not only experiencing a relatively larger second-level digital divide but also a "vulnerable" group among internal migrants concerning health. Furthermore, compared to IEMs with strong digital technology usage ability(50.98%), those with weak ability have a significantly lower proportion of self-rated "healthy"(40.84%), reflecting IEMs with strong digital technology usage ability have higher self-rated health status.

**Table 2:** Self-rated health status of IEMs (%).

Variables	Healthy	Basically healthy	Unhealthy	Disable
**Internal migrants**	82.18	15.12	2.61	0.10
**Internal elderly migrants**	42.74	38.51	17.58	1.16
with strong digital technology usage ability	50.98	37.90	10.61	0.51
with weak digital technology usage ability	40.84	40.16	17.87	1.12

### The overall impact and its dynamic changes

Expanding on Model (1), we employ *digital* to conduct ordered logit regression on *health*, to examine the overall impact of digital technology usage ability on IEMs’ health, as shown in columns (1). Moreover, in columns (2) and (3), we introduce the interaction terms digital*duration and digital*age to regress on *health*, respectively, to investigate the dynamic changes of digital technology usage ability affecting the health of IEMs over the duration of migration and with increasing age. The results are detailed in **Table 3**.

**Table 3:** Ordered logit regression results of the overall impact of digital technology usage ability on the health of IEMs and its dynamic changes.

Variables	(1) *health*	(2) *health*	(3) *health*
*digital*	0.3707*** (0.0737)	0.4114*** (0.1110)	2.4632*** (0.9181)
dgital*duration		-0.0045 (0.0091)	
digital*age			-0.0318** (0.0139)
*gender*	0.1013 (0.0697)	0.1015 (0.0698)	0.0972 (0.0698)
*age*	0.0546*** (0.0063)	0.0546*** (0.0063)	0.0792*** (0.0125)
*nation*	-0.1122 (0.1130)	-0.1117 (0.1130)	-0.1192 (0.1131)
*edu*	-0.2075*** (0.0349)	-0.2073*** (0.0349)	-0.2092*** (0.0349)
*hukou*	-0.2861*** (0.0780)	-0.2872*** (0.0781)	-0.2790*** (0.0782)
*marriage*	-0.0736 (0.0930)	-0.0735 (0.0930)	-0.0677 (0.0931)
*range_2*	0.1200 (0.0736)	0.1206 (0.0736)	0.1149 (0.0737)
*range_3*	0.4068*** (0.0842)	0.4087*** (0.0843)	0.4142*** (0.0843)
*duration*	0.0121*** (0.0039)	0.0155* (0.0079)	0.0119*** (0.0039)
*work*	0.7892*** (0.0794)	0.7894*** (0.0794)	0.7913*** (0.0795)
Simple N	3640	3640	3640
Pseudo R2	0.0553	0.0559	0.0550

Note: Coefficients are reported; standard errors are in parentheses.

*p<0.1

**p< 0.05

***p<0.001. The data in brackets is the standard error.

**Table 3** reveals that, in column (1), the coefficient of digital is significantly positive at a confidence level of 1%, indicating that IEMs with weak digital technology usage ability have a comparatively lower self-rated health status compared to those with strong ability. The empirical results generally support the negative impact of the digital divide on the health of IEMs.

In columns (2) and (3) of **Table 3**, the coefficients for digital are significantly positive at the 1% confidence level, while those for digital*duration and digital*age are negative, with only the latter being significant at the 5% confidence level. This implies that as migration duration and age increase, the adverse effects of digital technology usage ability on the health of IEMs tend to decrease. However, compared to migration duration, the negative health effects of the digital divide on IEMs exhibit a more pronounced decreasing trend with age.

### Heterogeneity of the impact in different cases

In order to explore the heterogeneity of the impact of the digital divide on IEMs’ health, we subdivided the samples of IEMs into different groups according to residence arrangement(reside), public health services(phservice), and medical insurance coverage(insurance). Subsequently, we conducted subsample regression of Model (1) for each group, with the regression results presented in **Table 4**.

**Table 4:** Results of heterogeneity test.

Variables	Residence arrangement (*reside*)		Public health services(*phservice*)		Medical insurance coverage (*insurance*)	
	Living alone or with a spouse only	Living with at least one offspring	Unreceived	Received	Uncovered	Covered
*digital*	0.3881*** (0.0863)	0.3233** (0.1427)	0.4596** (0.2011)	0.2732* (0.1560)	0.6053** (0.3072)	0.3559*** (0.0761)
Control variables	√	√	√	√	√	√
N	2647	993	544	758	225	3415
Pseudo R2	0.0558	0.0566	0.0633	0.0336	0.0596	0.0559

Note: The control variables involved in the estimation are the same as those in **Table 3**, but due to space limitations, here doesn’t report the estimated results of control variables

*p<0.1

**p< 0.05

***p<0.001.

**Table 4** shows that the coefficient of digital is significantly positive at the 10% confidence level in different sample regressions, indicating that the weaker digital technology usage ability has negative effects on the health of IEMs from different groups. From the perspective of residence arrangement, the sub-sample regression results of "living alone or with a spouse only" and "living with at least one offspring" show that the former’s coefficient of digital is slightly larger than that of the latter, indicating that the digital divide has a greater negative impact on the health of IEMs who live alone or only with a spouse. That is, support from younger family members can mitigate the adverse effects of the digital divide on IEMs’ health, but the reduction effect is not strong, perhaps because most of the youthful migrants have high work intensity and thus limited digital support for older people at home. In terms of public health services, the sub-sample regression results of "unreceived" and "received" show that the former’s coefficient of digital is obviously higher than that of the latter, indicating public health services can dramatically reduce the negative impact of the digital divide on the health of IEMs. Moreover, according to the magnitude of coefficient, public health services’ effect in mitigating the adverse impact of the digital divide is more than residence arrangement. Similarly, from the perspective of medical insurance coverage, the results show that the reduction effect of the groups with covered medical insurance is significantly higher than those with uncovered.

## Further research: Potential mechanisms test

### Social contact

With the rapid development of digital networks, various new media technologies have permeated every aspect of daily life, and it is increasingly difficult for people to escape the trend of digitization and media. Although the digital era has created a new and extensible spatial form for universal communication [19], as a digitally vulnerable group that was once far away from digital culture, IEMs, if unable to adapt to the interconnected lifestyle in the digital era, will face numerous obstacles in social contact, and may even be excluded from the system of digital social contact and become a digital island. Social contact among the migrant population is essential to accumulating social capital. Social capital can promote social mutual assistance, increase trust, enhance the sense of responsibility, and serve as an informal guarantee so that migrant populations can obtain mutual assistance from members of social networks when facing difficulties in the city. Moreover, social contact has also broadened the way to access information, including health knowledge and prevention of health risks, among migrant populations. Despite several empirical studies that have confirmed that social capital and access to information related to social contact can affect people’s health to some extent, it has also been pointed out that there are different types of social contact, each with unique traits and different impacts on health [20].

### Urban integration

The development of digital technology and its related network media has laid a deep foundation for forming a network society. For migrant populations, digital network media is not only a tool to communicate information and bond in a general sense, but also a social network behind it. People are increasingly completing online interactions and emotional and cultural exchanges through digital network media. Local governments are also paying more and more attention to online cultural images and conducting online cultural promotions. Through digital empowerment, IEMs can improve their ability to survive and develop in the city, solve practical difficulties, and then change their disadvantageous situation to better integrate into urban society [21]. Regarding the relationship between urban integration and health, some researchers believe that social integration can reduce the risk of death, improve mental health, and contribute to the establishment of healthy living habits [22,23]. Psychological integration is an advanced stage of urban integration for internal migrants, which can effectively improve their health [24,25]. However, some studies have pointed out that there are certain differences in the relationship between different dimensions of social integration and health, with three possible relationships being positive, negative, or curvilinear [26].

### Medical convenience

The integrated development of digital technology with healthcare services and industries has significantly enhanced the accessibility and convenience of information and services, thereby promoting the efficiency of health governance. Popularizing the basic knowledge of preventive healthcare via the Internet, implementing resident’s health management with intelligent means, assisting precise medical health services with digital technology, promoting the construction of medical insurance audit and real-time monitoring systems intelligently, and accelerating the improvement of public health warning systems through digital can effectively facilitate the overall level of health management, as well as improve the efficiency of medical and health services and reduce service costs, to bring medical convenience. More convenient and efficient medical insurance services are beneficial to people’s health. However, the effectiveness of digital in improving medical services efficiency is based on the premise that the service recipients can accept and effectively use these services. If they face barriers to the ability to use digital medical services, digitization will raise the threshold for people’s medical treatment and reduce access to medical services, consequently adverse to their health.

**Fig 1:**
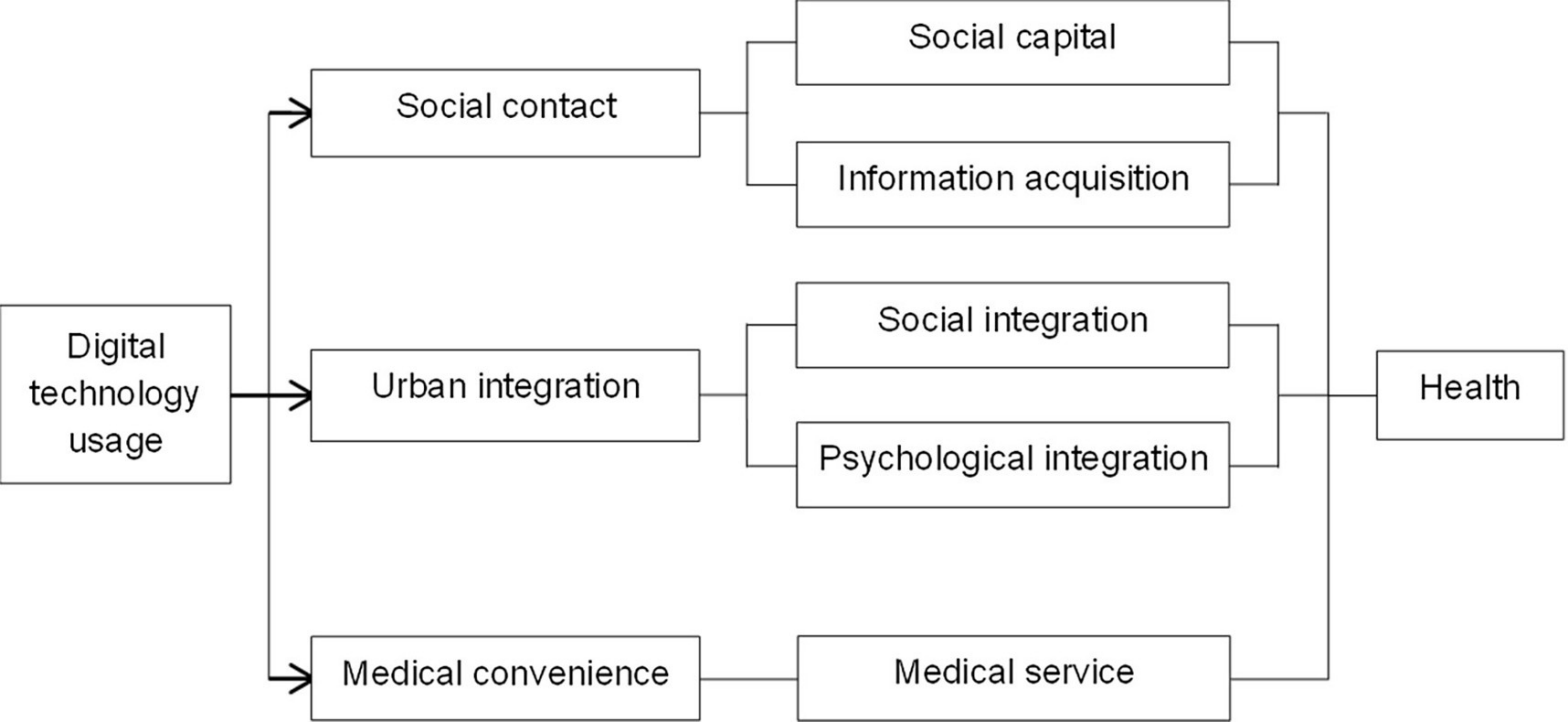
Mechanisms of the impact of digital technology usage ability on the health of IEMs.

Based on the above analysis, we believe that the digital divide may have different impacts on the health of IEMs through mechanisms such as social contact, urban integration, and medical convenience. Mechanisms by which digital technology usage ability affects the health of IEMs is illustrated in **Fig 1**.

We assess the social contact (contact)of IEMs by "how often contact with others", consider the urban integration(integration) by "self-perception that local residents whether willing to identify themselves as local people", judge the medical convenience(trouble) by "whether do not see a doctor due to inconvenience" to confirm the potential impact of the digital divide on the health of IEMs through the above three mechanisms. Thereby, we construct the mechanism variables i.e. contact, integration, and trouble, and turn to the three-step method standard procedure of mechanism test proposed by Baron and Kenny [27], testing whether each mechanism variable has mediating effects in the impact of the digital divide on the health of IEMs. The regression results for the first and third steps of the three-step method are shown in **Table 5**.

**Table 5:** Mechanisms test of the impact of the digital divide on the health of IEMs.

	Social contact		Urban integration		Medical convenience	
	(1)*contact*	(2)*health*	(3)*integration*	(4)*health*	(5)*trouble*	(6)*health*
*digital*	-0.2341*** (0.0833)	0.3570*** (0.0738)	-0.3552*** (0.0767)	0.3676*** (0.0740)	0.2901** (0.1279)	0.1901 (0.1219)
*contact*		-0.2678*** (0.0681)				
*integration*				-0.0272 (0.0525)		
*trouble*						0.3029*** (0.1027)
Control variables	√	√	√	√	√	√
N	3640	3640	3640	3640	1415	1415
Pseudo R2	0.0108	0.0573	0.0230	0.0553	0.0127	0.0501

Note: According to the nature of the dependent variables, except for columns (1) and (3) which use logit regression, the others all use ordered logit regression. The control variables involved in the estimation are the same as those in **Table 3**, but due to space limitations, here doesn’t report the estimated results of control variables.

*p<0.1

**p< 0.05

***p<0.001.

In the first step, when regressing the independent variable digital with three mechanism variables separately, all coefficients of digital are significant at the 1% confidence level. Moreover, from the sign of regression coefficients, we can see that stronger digital technology usage ability enhances the social contact of IEMs, improves their urban integration, and facilitates access to medical services. In the second step, when the digital technology usage ability variable digital is used to regress the self-rated health status variable health, the regression results in **Table 3** have confirmed that stronger digital technology usage ability has a positive impact on the health of IEMs. For the third step, we include the independent variable digital, dependent variable health, and mechanism variable contact (integration, trouble) in the same model. The regression results of columns (2) and (6) show that the coefficients for contact and trouble remain significant. Furthermore, compared with the coefficient of digital (0.3707) in the second step regression, when both digital and contact are regressed with the variable health, the coefficient of digital (still significant) only slightly decreased. However, when both digital and trouble are regressed with the variable health, the coefficient of digital becomes insignificant. Thus, according to Baron and Kenny’s mediating effects judgment criteria [27], urban integration does not have a mediating effect on the impact of the digital divide on the health of IEMs, social contact only has a partial mediating effect, and medical convenience has a significant mediating effect.

## Discussion

In this study, we use national elderly migrants-based data and apply the Grossman health demand model to assess the impact of the digital divide on the health among IEMs in China, to try to better facilitate their health improvement.

We find that the digital divide indeed affects the health of IEMs with adverse health outcomes, confirming the existence of the three-level digital divide. Previous studies have attributed digital technology usage can enable individuals, families, or social groups to get more up-to-date health information, access social support, adopt healthy behaviors, and make more informed medical decisions, ultimately achieving better health outcomes [28]. Therefore, the Chinese government has been actively promoting the use of ICT devices in medical services. However, individuals with varying characteristics stand to benefit differently from the utilization of digital technology. For instance, during the COVID-19 pandemic, panic caused by the epidemic has led to a flood of fake news, and those groups with low digital literacy may adopt detrimental epidemic prevention recommendations owing to their inability to discern the information’s authenticity, resulting in their poorer health [29]. The result gives us good inspiration: older folks, those with lower levels of education or income, and rural residents to being more susceptible to the negative effects of the three-level digital divide in terms of health [30]. IEMs not only confront the twin constraints of mobility and old age but also have generally lower levels of education and income, as well as limited digital technology usage ability. They inevitably become a group greatly affected by the digital divide that leads to adverse health outcomes. The results of our study seem to further confirm this.

Our results reveal that the negative impact of the digital divide on the health of IEMs will decrease with age. While the ability to use digital technology declines with age among IEMs, as this group’s inability to use it grows more common, the sense of exclusion experienced by those with weak digital skills in the digital society decreases, lessening the negative effects of the digital divide on their mental health. Another, when it becomes a consensus that older people have greater difficulty using digital devices, society proactively implement "aging-friendly" policies, such as decreasing the threshold for the elderly to use smart devices and prioritizing "offline" services. This will mitigate the adverse impact of the digital divide on IEMs’ access to health services. Consequently, the effects on physical and mental health suggest that the negative health effect of the digital divide on IEMs tends to decline with age.

The adverse effects of digital divide on the health of IEMs with different group characteristics are heterogeneous. Factors such as fear and negative attitudes towards digital technology usage and services, feeling too old to learn, lack of knowledge, and difficulty in understanding digital terminology are the main reasons of the digital divide among older people [31]. Cognitive and emotional support from family members not only helps older adults develop information literacy and learn digital skills but also helps them make better use of digital resources, which goes some way toward mitigating the negative effect of the digital divide. In addition to family support, social support also plays an essential role in bridging the digital divide. First, more "aging-friendly" offline public health services for digitally vulnerable IEMs can help them access more health information, knowledge, and skills, and more adequate medical services, contributing to better health outcomes. Second, medical insurance can ease the medical burden on IEMs when they see a doctor, as well as medical insurance agencies provide offline healthcare services to weakness and digitally vulnerable insured IEMs from time to time in order to reduce insurance payments, such as offline disease prevention campaigns, offline physical examinations, etc. These measures also reduce the adverse effect of the digital divide on insured IEMs’ health. Our results confirm that both family support (young family members) and social support (public health services and medical insurance) contribute to reducing the adverse effect of the digital divide on the health of IEMs, and social support plays a greater role in it.

Our paper further discusses potential mechanisms for the impact of the digital divide on the health of IEMs. Stronger digital technology usage ability of IEMs may enhance their social contact and urban integration, as well as improve medical convenience, all of which could have a positive impact on IEMs’ health. Our results reveal that the digital divide does not affect the health of IEMs through urban integration, only partially through social interaction, and mainly by affecting medical convenience.

What we investigate in this study is of significant policy implication. Our study confirms the existence of the three-level digital divide in IEMs from a health perspective. This means that in the context of the current rapid development of the digital economic, effective measures are needed to mitigate the adverse effects of the digital divide on the health of the digitally vulnerable IEMs to improve their overall health levels. First, enhance family support, that is, to encourage young family members to support IEMs with weak digital technology usage ability. Second, for the social aspect, which is to strengthen offline public health services for IEMs and expand their medical insurance coverage. In addition, considering that medical convenience is the primary mechanism by which the digital divide affects the health of IEMs, when medical and health institutions providing medical services, should not only focus on improving the level of digital services but also complement them with improved offline services for specific groups with weak digital technology usage ability. These approaches enable the full realization of the efficiency gains from digitization while mitigating the adverse effects of the digital divide, ensuring that all people can enjoy equal and convenient access to medical and health services.

The limitation of this study must be noted. Due to data limitations, we only used "whether getting health education through Community SMS/WeChat/Websites" as a measure of the digital technology usage ability of IEMs, and used this to determine whether they faced the digital divide. However, as digital technology becomes more widely used in the medical and health fields, the digital divide faced by the elderly will inevitably manifest in more dimensions. Therefore, in our future research, more comprehensive investigations and analyses are needed to fully understand the different impacts of various types of digital divides on the physical and mental health of the elderly, and to propose effective solutions, thereby providing recommendations for promoting the health and well-being of the elderly.

## Conclusion

In conclusion, this study shows that the digital divide does have an adverse effect on the health of IEMs, yet this effect diminishes with age. Our research also demonstrates the heterogeneity in the digital divide’s impact on the health of various subgroups of IEMs, which should be taken into account by policies. Furthermore, the digital divide primarily affects the health of IEMs through its influence on medical convenience. Therefore, the government is urged to regard the IEMs with vulnerable characteristics in digital technology usage as the key population in the future digital equalization policies and take targeted measures to improve their offline medical convenience.

## Supporting information

S1 File Empirical data in STATA. (RAR)

S2 File Do file in STATA. (RAR)
